# The Ephb2 Receptor Uses Homotypic, Head-to-Tail Interactions within Its Ectodomain as an Autoinhibitory Control Mechanism

**DOI:** 10.3390/ijms221910473

**Published:** 2021-09-28

**Authors:** Yan Xu, Dorothea Robev, Nayanendu Saha, Bingcheng Wang, Matthew B. Dalva, Kai Xu, Juha P. Himanen, Dimitar B. Nikolov

**Affiliations:** 1 Department of Veterinary Biosciences, College of Veterinary Medicine, The Ohio State University, Columbus, OH 43210, USA; xu.4695@osu.edu (Y.X.); xu.4692@osu.edu (K.X.); 2 Structural Biology Program, Memorial Sloan-Kettering Cancer Center, 1275 York Avenue, New York, NY 10065, USA; doroty17@yahoo.com (D.R.); sahan@mskcc.org (N.S.); 3 Rammelkamp Center for Research, MetroHealth Medical Center, 2500 MetroHealth Drive, Cleveland, OH 44109, USA; bxw14@case.edu; 4 Department of Neuroscience and Jefferson Center for Synaptic Biology, Thomas Jefferson University, 233 South 10th Street, Bluemle Life Sciences Building, Room 324, Philadelphia, PA 19107, USA; matthew.dalva@jefferson.edu

**Keywords:** receptor tyrosine kinases (RTKs), Eph receptors, ligand-binding domain, fibronectin type III domain, X-ray crystallography, receptor clusters, protein–protein interfaces, kinase activation

## Abstract

The Eph receptor tyrosine kinases and their ephrin ligands direct axon pathfinding and neuronal cell migration, as well as mediate many other cell–cell communication events. Their dysfunctional signaling has been shown to lead to various diseases, including cancer. The Ephs and ephrins both localize to the plasma membrane and, upon cell–cell contact, form extensive signaling assemblies at the contact sites. The Ephs and the ephrins are divided into A and B subclasses based on their sequence conservation and affinities for each other. The molecular details of Eph–ephrin recognition have been previously revealed and it has been documented that ephrin binding induces higher-order Eph assemblies, which are essential for full biological activity, via multiple, distinct Eph–Eph interfaces. One Eph–Eph interface type is characterized by a homotypic, head-to-tail interaction between the ligand-binding and the fibronectin domains of two adjacent Eph molecules. While the previous Eph ectodomain structural studies were focused on A class receptors, we now report the crystal structure of the full ectodomain of EphB2, revealing distinct and unique head-to-tail receptor–receptor interactions. The EphB2 structure and structure-based mutagenesis document that EphB2 uses the head-to-tail interactions as a novel autoinhibitory control mechanism for regulating downstream signaling and that these interactions can be modulated by posttranslational modifications.

## 1. Introduction

Eph receptors constitute the largest family of receptor tyrosine kinases (RTKs) [1]. They, and their membrane-anchored ephrin ligands, are divided in two subgroups, A and B, based on binding specificities and sequence conservation [2]. A and B class ephrins also differ by the mode of membrane attachment: The A class ephrins have a GPI (glycosylphosphatidylinositol) glycolipid anchor, while the B class ephrins have a transmembrane sequence and a small cytoplasmic domain [3]. An important characteristic of these molecules is that, upon cell–cell contact, signals are transduced into both the Eph- and ephrin-bearing cells [4,5]. With few exceptions, A class receptors bind A class ligands, whereas B class receptors bind B class ligands [6]. The Ephs and ephrins were originally characterized as mediating axon guidance, but their roles in various other cell–cell-communication events have also been documented [7,8]. Their role in tumor development and cancer progression has lately been intensively investigated [9,10,11]. In particular, EphB2 signaling regulates many developmental processes and adult tissue homeostasis [12]. It enhances tumor proliferation through a kinase-dependent pathway but also inhibits cell migration independent of its kinase activity [13]. Moreover, it has been shown that Alzheimer disease-linked amyloid-β oligomers bind to the fibronectin domains of EphB2 and trigger receptor degradation in the proteasome [14]. Thus, augmenting EphB2 activity could have beneficial effects in Alzheimer disease by reversing long-term potentiation impairments [15]. Therapeutic strategies targeting amyloid-β oligomers were recently reviewed in [16].

The Eph extracellular region or ectodomain (ECD) is a multidomain assembly, consisting of a ligand-binding domain (LBD), a cysteine-rich domain (CRD), and two fibronectin (FN) III domains (FN1 and FN2) [17]. Ephrins, on the other hand, have only one domain on the outside of the cell, the receptor-binding domain (RBD). Earlier structural studies with the minimal ligand-receptor binding domains revealed the molecular details of the Eph–ephrin recognition [18,19,20,21]. Thus, upon binding, a long hydrophobic loop of the ligand inserts into a hydrophobic cavity on the surface of the receptor. While this interaction offers the energetic driving force for the binding and is necessary and sufficient for the formation of heterodimeric Eph–ephrin complexes, it is not sufficient to cause the activation of the receptors on the cell surface [22]. Indeed, imaging studies with Eph-expressing cells have shown that, unlike the ‘canonical’ RTKs, where simply bringing two receptors close to each other is enough for signaling [23], the Eph receptors require the formation of higher-order assemblies or clusters, for full biological activity [24,25]. 

Structures of entire Eph ECDs bound to ephrins revealed that their clustering requires two separate receptor–receptor interfaces [26,27,28], both of which have neighboring Eph molecules interacting in a head-to-head (parallel) orientation. The first interface involves the Eph LBD region but can facilitate bringing two Eph molecules together even in the absence of ligand. It was originally named ‘Eph–Eph homo-dimerization’ interface. The second distinct and non-overlapping interface is located within the CRD and was named ‘Eph clustering’ interface. Each of the unoccupied clustering interfaces within an Eph dimer can work independently to recruit another receptor dimer in the assembly. This process can then proceed until a fully active Eph cluster, consisting of perhaps hundreds of receptors, is formed. A fascinating conclusion from these studies is that signaling-competent clusters may form once the local receptor concentration is high enough to allow the utilization of both head-to-head Eph–Eph interfaces [29,30]. This can happen even in the absence of ligand, provided the Eph expression levels are high enough, as observed in certain cancer cells [31]. 

Earlier studies on unliganded Eph ECDs revealed the existence of yet another, third, Eph–Eph interacting interface, this time with head-to-tail orientation of the interacting Ephs, formed between the LBD and the FN domains of two adjacent unliganded Eph molecule [32,33]. Once an Eph receptor binds a ligand, this homotypic Eph–Eph interaction falls apart, displaced by the Eph–ephrin interaction. The exact biological relevance of this interface is still under scrutiny, but it has been suggested to be involved in fine-tuning the Eph signaling. For example, it could potentially collaborate with Eph–ephrin in-*cis* interactions [34] that have been reported for certain cell types, to more precisely control the levels of Eph kinase phosphorylation [35]. The Eph–Eph head-to-tail homotypic interactions might also be responsible for two intriguing Eph signaling phenomena: First, the Eph clusters sometimes seem to cover larger cell surface areas than what would be expected based on the direct Eph–ephrin contact areas [36]; second, unliganded (containing mutations abolishing ligand binding) Eph receptors can be recruited into the pre-existing Eph–ephrin clusters [37,38]. To further investigate the significance of the receptor–receptor homotypic interactions, particularly in the B class Ephs, we crystallized the EphB2 ECD, determined its three-dimensional structure, and performed structure-based mutagenesis and Eph kinase activation assays. The reported data reveal that Eph receptors use receptor–receptor interactions as a unique autoinhibitory mechanism to control Eph signaling.

## 2. Results and Discussion

**Figure 1:**
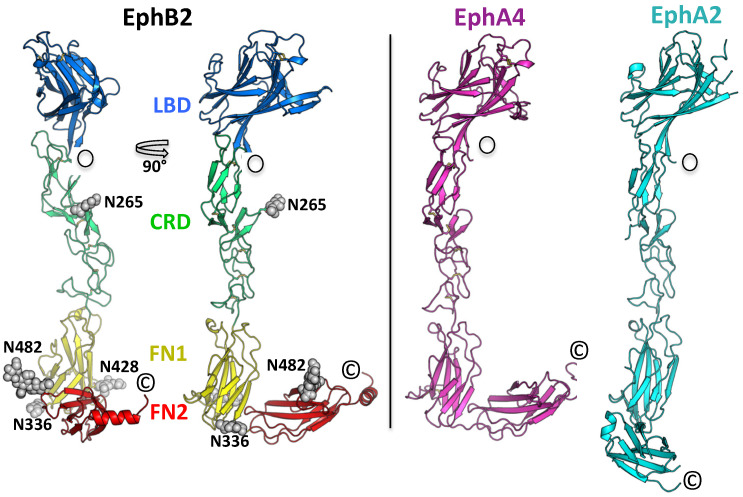
(**Left**) Crystal structure of the EphB2–ECD. Two orthogonal views are shown. The protein has an elongated architecture (~150 Å long and 25–50 Å wide) and all four domains are well-ordered. The LBD–CRD–FN1 segment is rigid, while the connection between the FN1 and FN2 domains is flexible. The individual EphB2 domains are colored differently, and the attached glycans are in gray. (**Right**) Comparison of the EphB2 ECD structure with the other two reported full ECD structures, EphA4, and EphA2. Only the positioning of the second FNIII domain is distinct in these structures, consistent with the flexibility of the FN1–FN2 linker region.

The mouse EphB2 extracellular domain (ECD) (residues 19–543) was purified using Protein A Sepharose chromatography and size-exclusion chromatography. Size-exclusion chromatography and pull-down assays (data not shown) documented that the protein was properly folded and bound ephrin ligands with high affinity. Its identity and purity were confirmed by mass spectrometry. The protein was crystallized in 1.1 M Na Succinate, 0.1 M Na Acetate pH 4.8, 5% (*v*/*v*) MPD, and 3% 1, 6-Hexanediol, and the structure was determined by molecular replacement at 3.1 Å resolution with a final R-free of 25%. The refinement statistics are given in Table 1. Overall, the protein shows an extended, elongated architecture (~150 Å long and 25–50 Å wide) with all four domains well ordered (Figure 1). The CRD can be subdivided in two domains: The N-terminal one resembles the complement regulator domain (sometimes called a ‘Sushi’ domain) and is followed by an epidermal growth factor (EGF)-like domain. As in previously published Eph–ECD structures [27,28,32], the region containing the LBD, the CRD, and FN1 is rigid, but the connection between FN1 and FN2 is flexible. Indeed, in the EphA2–ECD and the EphB6–ECD structures, the second FN domain is not even visible in the electron density map [27,39]. The average r.m.s.d. between the Cα positions of the EphB2 and EphA2 ectodomains, excluding FN2 (over 367 Cα atoms), is 2.6 Å, and that between the EphB2 and EphA4 entire ectodomains (over 465 Cα atoms) is also 2.6 Å. The comparison of the three known Eph full-ectodomain structures is shown in Figure 1. The EphB2 protein is glycosylated at four sites: N265, N336, N428, and N482, as illustrated. 

**Figure 2:**
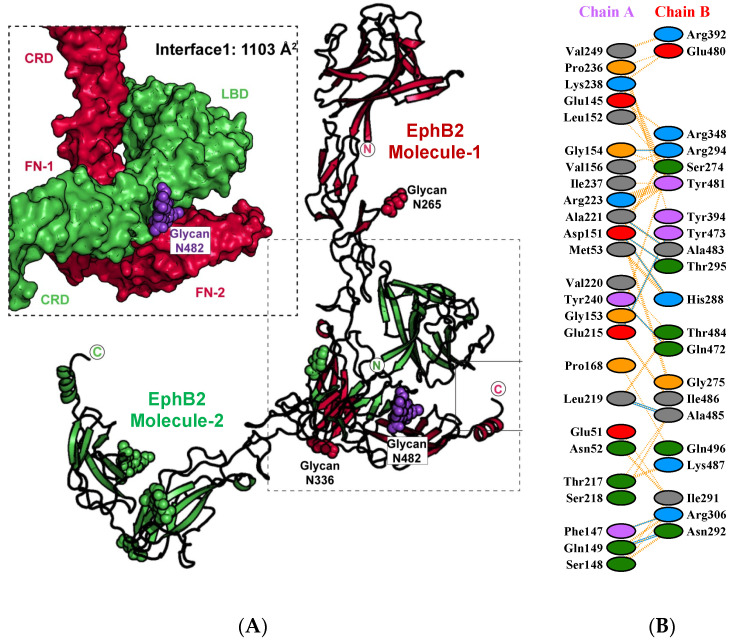
The head-to-tail EphB2 interface. (**A**) The two adjacent, interacting Eph molecules are colored in red and green. The glycan attached to residue Asn-482 is in purple. The zoom-in on the left shows a space-filling model of the interface formed between the LBD and the FNIII domains of the two interacting EphB2 molecules. (**B**) Schematic diagram of the EphB2–ECD residues interacting within the head-to-tail interface. Residue color: positive, blue; negative, red; neutral, green; aliphatic, gray; aromatic, magenta; P,G, orange.

As in some of the previously reported unliganded Eph ectodomain structures, the EphB2–ECD forms homotypic head-to-tail dimers via interactions between the LBD and the FNIII domains of two neighboring molecules (Figure 2A). The size of the LBD–FN interface is 1103 Å^2^ and the specific interacting residues are listed in Figure 2B. The total interacting surface area is about the same size as the area observed in the EphA2–ECD structure [27] (980 Å^2^) but smaller than the one observed in the EphA4–ECD structure [32] (2460 Å^2^). It was suggested [33] that the ligand promiscuity of EphA4 might, in part, be a result of its larger LBD–FN interface that would ensure a faster and more efficient receptor activation as compared to the other Eph receptors. Specifically, it was proposed that, while the head-to-head Eph–Eph interfaces are responsible for ligand-induced Eph clustering, the head-to-tail interface is responsible for Eph pre-clustering prior to ligand binding. Thus, the head-to-tail FN–LBD receptor–receptor interactions are emerging as a new paradigm for explaining the unique regulation and fine-tuning of Eph–ephrin signaling. Indeed, similar head-to-tail interactions have not been reported for other RTKs, while their biological importance for Eph signaling is further underscored by the existence of several cancer-related mutations within the FNIII domains, in addition to those within the LBD and CRD [40].

Although the Eph–Eph head-to-tail interactions in the EphB2 structure have some similarities with those observed in the EphA2 and EphA4 structures, there are also major differences. For example, the FN C-terminal ‘tail’ (FN1-FN2) of one EphB2 molecule wraps around the LBD ‘head’ of the other molecule, covering several residues beyond the LBD and the FN2 domains (Figure 2A). In contrast, all interacting residues in the A class structures reside exclusively within the LBD and the FN2 domains. In EphB2, in addition to the 13 LBD and 10 FN2 residues, there are 12 residues within the CRD (including Ile-291, Asn-292, Arg-294, and Thr-295) and three residues within the FN1 (Arg-348, Arg-392, and Tyr-394) that participate in the head-to-tail interactions (Figure 2B). Of these, Arg-348 seems of particular importance because it forms a salt bridge with Glu-145 of the LBD, a residue that is adjacent to the surface involved in ligand binding. While Glu-145 is conserved in both A and B class receptors, Arg-348 is unique to EphB1, EphB2, and EphB3. Hence, the resting-state EphB receptors could need a higher concentration of engaging ligand to outcompete the LBD–FN interactions as compared to their A class counterparts. Indeed, the A class receptors have been reported, in general, to form ephrin complexes with faster on rates and lower Kds [41].

Interestingly, the glycosylated Asn-482 in the FN2 domain is in the heart of the head-to-tail interface, revealing its important role in the homotypic Eph–Eph interactions. As illustrated on Figure 2A, the Asn-482-attached glycan wraps around the connection between the LBD and CRD of the interacting molecule, locking in place the LBD and CRD of one Eph molecule against the FN1–FN2 region of its neighbor. The N482-attached glycan forms a hydrogen bond with the Arg-223 side chain. Of the other three glycosylated residues, only Asn-336 is close to the head-to-tail interface, but still the Asn-336-attached glycan is at least 12 Å from the interacting Eph molecule. Glycan moieties have been previously shown to have roles in mediating other protein–protein interactions, for example, in inflammation and immunity [42,43]. While it has been shown that the glycosylation status of the ephrins is important for signaling and proliferation of glioblastoma cells [44], there have been no prior reports on the role of Eph receptor glycosylation in Eph signaling. For example, earlier structural studies of the EphA2 ECD documented that the two putative N-linked glycosylation sites, located in the FN1 domain and in the linker between FN1 and FN2, are not involved in the EphA2 homotypic contacts [28]. Indeed, the Asn-482 glycosylation site is only found in EphB1 and EphB2 and not in any other human A or B class receptors (Table 2), with most A class receptors containing either a Ser or a Thr residue at the corresponding position [27,32].

**Figure 3:**
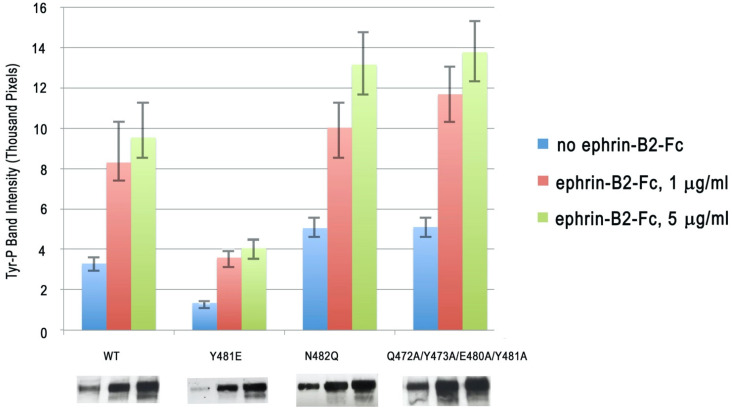
EphB2 kinase phosphorylation of the wild-type receptors and receptors harboring mutations in the head-to-tail interface. One of the mutants (Y481E) was designed to electrostatically stabilize the LBD–FN2 interaction by a formation of a salt bridge with Arg-223 in the LBD. Two other mutations were designed to disrupt the LBD–FN2 interaction, either by abolishing the glycosylation site (N482Q) or by disrupting key hydrogen and van der Waals bonds (Q472A/Y473A/E480A/Y481A) at the interface. The stabilizing mutation inhibited EphB2 phosphorylation and signaling, while both destabilizing mutations increased EphB2 phosphorylation. Blue, constitutive signal with no added ephrin ligand; red, 1 μg/mL ephrin-B2; green, 5 μg/mL ephrin-B2. Representative Western blot scans are shown under the labels.

In order to study the importance of the EphB2 glycosylation and the head-to-tail EphB2 homotypic interactions, we generated two mutations (a single point mutation and a quadruple mutation) to disrupt the LBD–FN interactions. The first mutation, N482Q, abolished the glycosylation site at the LBD–FN2 interface, while the second mutation, Q472A/Y473A/E480A/Y481A, disrupted key hydrogen and van der Waals bonds at the head-to-tail interface. We introduced these mutations into the full-length EphB2 receptor, and the engineered constructs were transfected in HEK293 cells. EphB2 activation was monitored by measuring the level of the EphB2 phosphorylation after ephrin stimulation. As shown in Figure 3, both of these destabilizing mutations facilitated receptor kinase activation. The intensity of the phosphorylated EphB2 band on a protein gel was approximately 40% and 46% higher for the N482Q and the Q472A/Y473A/E480A/Y481A mutations, respectively, as compared to the wild-type receptor. The increase in the phosphorylation over wild-type EphB2 was statistically significant (*p* < 0.05) for both mutations, while the difference between the two mutations was not statistically significant. 

**Figure 4:**
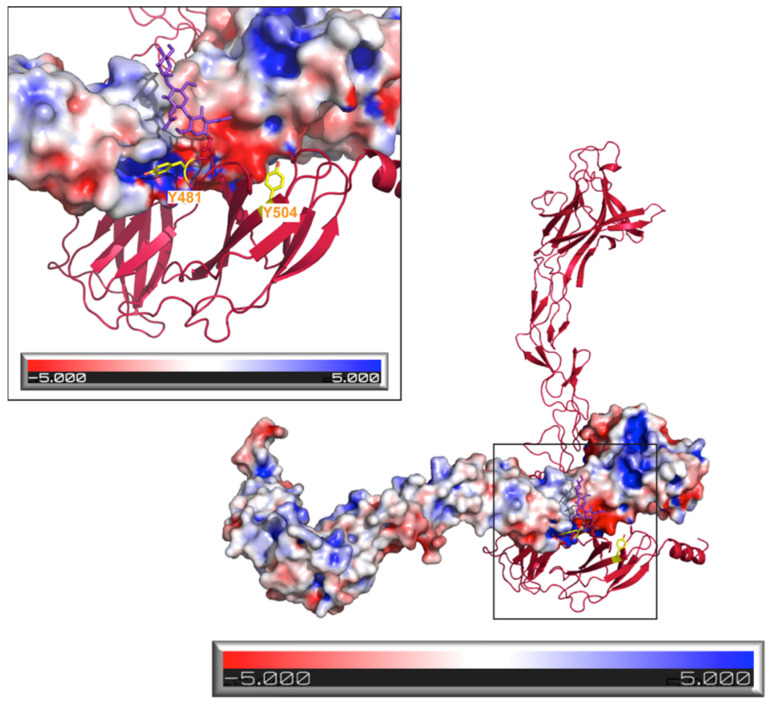
Electrostatic surface potential of EphB2 with a zoom-in at the head-to-tail interface. Tyrosines at positions 481 and 504 are shown in yellow.

We next generated a mutation, Y481E, which was designed to electrostatically stabilize the EphB2 head-to-tail interaction. As illustrated on Figure 4, which shows the electrostatic surface potential of EphB2, Tyr-481 interacted with a highly positively charged LBD area in the head-to-tail dimers and the Y481E mutation was designed to allow the formation of a new E481-R223 salt bridge. The cell-based Eph activation assays reveal that this stabilizing mutation (Y481E) decreased receptor activation and signaling, as illustrated by an approximate 56% lower EphB2 kinase domain phosphorylation when compared to the wild-type receptor (Figure 3). 

The selection of the Y481E mutation was also based on a recent report [45] that the extracellular region of EphB2 can be phosphorylated in vivo. The two tyrosine residues found to be phosphorylated under certain conditions were Tyr-481 and Tyr-504. While Tyr-504 was not a part of the homotypic Eph–Eph interface (but was located relatively close to it, ~10 Å), Tyr-481 was at the heart of the interface (Figure 4). Our mutagenesis results were consistent with the notion that phosphorylation of Tyr-481, which was mimicked by the Y481E mutation, could be used to regulate (in this case, suppress) the autoinhibitory EphB2 interactions, thus modulating the signaling. The head-to-tail EphB2 interactions would also interfere with access to both Tyr-481 and Tyr-504 of potential tyrosine kinases, consistent with the observation that ligand binding facilitates EphB2 ECD phosphorylation [45]. Notably, since EphB2 ECD phosphorylation has been shown to regulate the EphB2-NMDAR interactions [45], our results suggest that the EphB2 head-to-tail interactions might modulate NMDAR signaling. Interestingly, Tyr-481 is unique to EphB2, underscoring the possibly unique regulation of the EphB2 homotypic autoinhibitory interactions, and thus EphB2 signaling, via ECD phosphorylation. 

**Figure 5:**
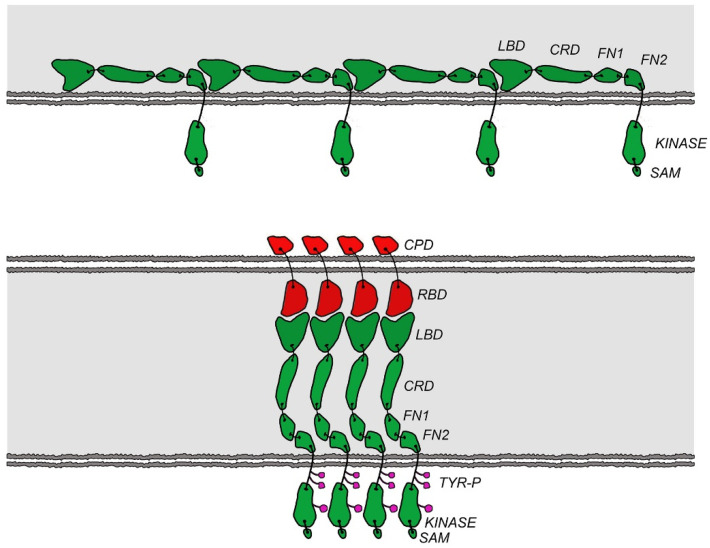
Schematic representation of the autoinhibitory regulation mechanism of EphB2. (**Upper panel**) In the unliganded receptors (green), the head-to-tail LBD–FN interactions kept the kinase domains unphosphorylated and inactive by separating neighboring receptors (~150 Å apart). (**Lower panel**) Upon ligand (red) binding, the LBD–FN interactions were displaced by the LBD–ephrin interactions. The receptors moved closer to each other, utilizing the head-to-head Eph–Eph interfaces within the LBD and CRD, thus causing receptor trans-phosphorylation on juxtamembrane and kinase domain tyrosines. LBD, ligand-binding domain; CRD, cysteine-rich domain; FN, fibronectin; SAM, sterile alpha motif; Tyr-P, phosphorylated tyrosine residue; CPD, cytoplasmic domain; RBD, receptor-binding domain.

Our results showed for the first time that EphB receptors can use head-to-tail homotypic interactions as an autoinhibitory mechanism. Importantly, the three EphB2–ECD mutations discussed above affected both the constitutive (ligand-independent) and the ligand-induced EphB2 phosphorylation (Figure 3). The mutations that disrupted the head-to-tail (LBD–FN) EphB2 interface increased EphB2 phosphorylation, while the mutation that stabilized the interface decreased it. The simplest explanation for the autoinhibitory effect of the LBD–FN interactions is that they positioned the receptors on the cell surface at a distance from one another that was larger than the distance required for efficient trans-phosphorylation of neighboring molecules (Figure 5). Indeed, the EphB2 ECD structure showed that the receptor ectodomain was a rigid rod, with a hinge between the FN1 and FN2 domains. When the N-terminal LBD of one EphB2 molecule interacted with the membrane-proximal FN region of its neighbor, the two molecules were separated by the long, rigid LBD-CRD-FN1 region that was approximately ~150 Å long. Upon ligand binding, the LBD–ephrin interactions would displace the LBD–FN head-to-tail interactions, and the LBD would move away from the cell membrane, towards the ephrin-expressing cell. The Eph ECD would now be an extended rod that was approximately perpendicular to the membrane and involved in both Eph–ephrin and Eph–Eph head-to-head interactions, as reported for the liganded EphA2 and EphA4 receptors [32,33]. Consecutively, the distance between the transmembrane domains of neighboring receptors would decrease from ~150 Å to a much closer arrangement (Figure 5), as seen in the EphA–ephrinA complex structures. 

Interestingly, the previously reported head-to-tail LBD–FN interactions in the EphA4 receptor were not autoinhibitory. On the contrary, mutations that disrupted these interactions tended to inhibit EphA4 signaling, while mutations that stabilized the interactions facilitated receptor phosphorylation [32]. Therefore, it was suggested that the primary role of the head-to-tail interactions in EphA4 is to pre-cluster the receptors in the absence of ligand, which would facilitate the ligand-induced receptor activation. The data on EphB2 reported here showed that most likely the A and the B class receptors, and certainly EphB2 and EphA4, dramatically differed in how they used the head-to-tail receptor–receptor interactions to modulate cell-cell signaling. The two distinct regulatory mechanisms (autoinhibition and pre-clustering) are consistent with observations that A class Eph receptors, in general, have higher affinity for their cognate ligands than the B class receptors and can undergo activation at lower ligand concentrations [41]. As mentioned above, another stark contrast between A and B class Eph–Eph head-to-tail interactions is that in EphB2 they are dependent on (and could potentially be regulated by) post-translational receptor modifications, such as phosphorylation and glycosylation, unlike EphA2 and EphA4. This suggests that Eph B class signaling might be under a tighter regulatory control than Eph A class signaling. 

Finally, the fact that homotypic LBD–FN interactions have now been shown to regulate both A and B class Eph signaling identifies the Eph FN region as a potential drug target. Several monoclonal antibodies raised against LBDs of both A and B Eph classes, including EphB2, are currently in preclinical and clinical development [46,47,48,49] and it would be interesting to also generate and characterize antibodies targeting the LBD-interacting FN regions of these molecules. 

## 3. Materials and Methods

### 3.1. Cloning and Mutagenesis

Mouse EphB2–ECD (residues 19–543) was cloned into a modified pAcGP67 baculovirus expression vector (BD Bioscience, Becton Dickinson Franklin Lakes Campus, NJ, USA), with GP67 secretion signal and human Fc fragment as C-terminal tag. Recombinant baculovirus was generated by co-transfecting the expression plasmid along with linearized BaculoGold DNA (BD Pharmingen Inc, San Diego, CA, USA) into SF9 cells. The human EphB2 full-length protein and the structure-based mutants were cloned into a pcDNA3.1 + hygromycin vector (Invitrogen, Waltham, MA, USA) for stable expression in a HEK293 cell line. Mutations were introduced by site-directed mutagenesis (Stratagene, San Diego, CA, USA) and were sequence-verified. 

### 3.2. Protein Expression and Crystallization

Hi5 cells were infected with passage 3 baculoviruses (for EphB2-ECD expression) at a multiplicity of infection of 10. The medium containing the secreted fusion protein was collected 72 h postinfection and loaded onto a Protein A-Sepharose affinity column. Recombinant protein was eluted with low-pH buffer containing 150 mM NaCl and 100 mM glycine (pH 3.0). The Fc tag was cleaved by thrombin proteolysis and removed by Protein A-Sepharose. EphB2-ECD was further purified by gel filtration chromatography on a Superdex 200 column. The purified protein was concentrated to 10 mg/mL in Hepes buffered saline (HBS) and crystallized by hanging-drop vapor diffusion at room temperature against a well solution of 1.1 M Na Succinate, 0.1 M Na Acetate pH 4.8, 5% (*v*/*v*) MPD, and 3% 1, 6-Hexanediol. For data collection, the crystals were frozen in a cryo-buffer containing an additional 25% (*vol/vol*) glycerol. X-ray diffraction data were collected at beamline 24ID-C (Northeastern Collaborative Access Team, Advanced Photon Source) and processed with HKL2000 [50]. The structure was determined by molecular replacement with EphA2 [Protein Data Bank (PDB) ID is 3FL7] [27] as a search template, using the Phaser program [51] in the Phenix suite [52]. Subsequent refinement proceeded with iterative rounds of model adjustments, using the programs Coot [53] and PhenixRefine [52]. Crystallographic details and statistics are listed in Table 1.

### 3.3. Cell Manipulations and Transfections

HEK293 cells were grown in DMEM (Invitrogen) supplemented with 10% (*vol/vol*) FBS, 100 units/mL penicillin, and 100 μg/mL streptomycin. Cells were consistently transfected at 80–90% confluence in six-well plates using Lipofectamine 2000 (Invitrogen). 

### 3.4. Cell-Based EphB2 Kinase Activation Assay

The EphB2 activation assays were performed using a previously reported method [32]. HEK293 cells were stably transfected with full-length wild-type EphB2 or EphB2-containing mutations in the head-to-tail interface (Y481E, N482Q, and Q472A/Y473A/E480A/Y481A). Clones with similar expression levels, tested by anti-EphB2 antibody in Western blots, were chosen for the assay. EphB2-expressing cells were then challenged with ephrin-B2-Fc, which was clustered using anti-human IgG antibody at a 25-nM concentration. After 10 min of incubation with ligand, cells were washed with PBS and harvested. Total cell lysate was prepared by lysing cell pellets in a buffer containing 20 mM Hepes (pH 7.4), 150 mM NaCl, 1% (*wt/vol*) Nonidet P-40, and 1 mM EDTA. Activated receptor was immunoprecipitated with anti-phosphotyrosine antibody (Upstate Biotechnology) and Protein A-Sepharose beads, resolved on SDS-PAGE, and blotted onto PVDF. Membranes were then blotted with anti-EphB2 antibody (R&D Biotechnology). The Western blot films were scanned and quantified by spot densitometry using ImageQuantTL software 7.0 (GE Healthcare Biosciences, Chicago, IL, USA). All intensity readings were normalized for the EphB2 amount in total cell lysate and calculated as the ratio to the wild-type (WT) basal activity (ligand-independent phosphorylation level). The experiments were done in triplicate and the error bars in Figure 3 represent SD. 

### 3.5. Illustrations

Figures were prepared using Adobe Illustrator (Adobe) and Photoshop. All molecular representations were produced with PyMOL. 

## Figures and Tables

**Table 1:** Data collection and refinement statistics for the EphB2 ECD crystal structure. Statistics for the highest-resolution shell are shown in parentheses.

	EphB2-ECD (PDB ID: 7S7K)
Resolution range (Å)	48.4–3.14 (3.32–3.14)
Space group	P 2_1_ 2_1_ 2_1_
Unit cell	73.843 111.142 156.877 90 90 90
Total reflections	73,799
Unique reflections	22,498
Multiplicity	3.3 (3.4)
Completeness (%)	97.26 (98.52)
Mean I/Sigma(I)	18 (1.5)
Wilson B-factor	116.94
R-merge	0.041 (0.792)
R-work	0.1913 (0.3053)
R-free	0.2469 (0.3427)
Number of atoms	4167
*Macromolecules*	4072
*Ligands*	95
*Water*	0
Protein residues	532
RMS (bonds)	0.010
RMS (angles)	1.43
Ramachandran favoured (%)	95
Ramachandran outliers (%)	0.19
Clash-score	12.64
Average B-factor	48.50
*Macromolecules*	47.10
*Ligands*	109.90

**Table 2:** Alignment of Eph sequences around the N482 glycosylation site (magenta) of EphB2.

h-EphB1	(469) iryyekehnefnssm-ar (485)
h-EphB2	(471) lqyyekelseynata-ik (487)
h-EphB3	(488) mkyfek--segiast-vt (502)
h-EphB4	(443) vkyhekgaegpssvrflk (460)
h-EphB6	(508) lryydqaedeshsftmts (525)
h-EphA1	(469) vkyhekgaegpssv-vle (485)
h-EphA2	(443) vtyrkkgdsnsynv-rrt (459)
h-EphA3	(472) vkyyekqeqetsyti-lr (488)
h-EphA4	(476) vkyyekdqnersyri-vr (492)
h-EphA5	(504) ikyfekdq-etsyti-ik (519)
h-EphA6	(477) tkyyekeheqltyss-tr (493)
h-EphA7	(443) ikyyekdqrertyst-lk (459)
h-EphA8	(475) ikyyekdkemqsyst-lk (491)
h-EphA10	(492) iryyekgqseqtysmvkt (509)
EphB2	
Human	(471) lqyyekelseynat
Mouse	(471) lqyyekelseynat
Rat	(471) lqyyekelseynat
Chicken	(479) lqyyeknlselnst
Macaque	(448) lqyyekelseynat

## Data Availability

The EphB2–ECD structure coordinates and structure factors are deposited in the RCSB Protein Data Bank with accession code 7S7K.
